# Effect of Transgenic Cotton with Bt Event Mpp51Aa2 on Cotton Fleahopper (*Pseudatomoscelis seriatus*) During Early Cotton Growth and Resulting Plant Injury

**DOI:** 10.3390/insects17030233

**Published:** 2026-02-24

**Authors:** Malek A. Alwedyan, Pius A. Bradicich, Joshua A. McGinty, Michael J. Brewer

**Affiliations:** Texas A&M AgriLife Research and Extension Center, Corpus Christi, TX 78406, USA; malekabd84@yahoo.com (M.A.A.);

**Keywords:** *Gossypium hirsutum*, ThryvOn trait (Mpp51Aa2; Cry51Aa2), plant injury, yield loss, plant bugs

## Abstract

The cotton fleahopper (*Pseudatomoscelis seriatus* (Reuter)) is an early-season hemipteran plant bug pest of cotton. In transgenic cotton cultivars, Bt event Mpp51Aa2 (Cry51Aa2) affects some other plant bug species (Hemiptera: Miridae) (we henceforth use the common terminology, stating that these cultivars have the ThryvOn trait). Field experiments were conducted in Corpus Christi, Texas, USA, to evaluate the responses of four cotton cultivars with and without the ThryvOn trait to cotton fleahopper infestations, comparing them with early-season foliar insecticide applications of flupyradifurone. Naturally occurring cotton fleahopper populations rose to the economic threshold midway through the first four weeks of squaring, when cotton plants are most sensitive to feeding injury, in 2024 and later in the same period in 2025. Flupyradifurone application significantly reduced adult and nymph abundance, resulting in reduced square abscission (2025) and yield protection that was consistent across cultivars in 2024 but more variable in 2025. Cultivars expressing the ThryvOn trait exhibited modest and variable reductions in adult cotton fleahopper abundance (~10–30%), occasionally benefiting from reduced square abscission but not protected yield. The open boll ratio, used to assess maturity delay, was not affected by cultivar or cotton fleahopper pressure. These results indicate that ThryvOn cultivars may provide limited supplemental benefits but do not replace or enhance the effectiveness of flupyradifurone for cotton fleahopper management.

## Simple Summary

The cotton fleahopper is an important early-season hemipteran pest of cotton. Transgenic cultivars expressing the ThryvOn trait affect some plant bug species. Field experiments conducted in Corpus Christi, Texas, USA, evaluated the responses of cotton cultivars with and without the ThryvOn trait to cotton fleahopper infestations and early-season insecticide application (flupyradifurone). Cotton fleahopper populations reached the economic threshold midway through the first four weeks of squaring, when cotton plants are most sensitive to feeding injury, in 2024 and later in the same period in 2025. Flupyradifurone application reduced adult and nymph cotton fleahopper populations, reduced square loss, and protected yield across cultivars. Cultivars expressing the ThryvOn trait exhibited modest and variable reductions in adult cotton fleahopper abundance (~10–30%) and did not provide consistent yield protection. Cotton maturity was not affected. These results indicate that ThryvOn cultivars may provide limited supplemental benefits but cannot replace insecticide-based cotton fleahopper management.

## 1. Introduction

The cotton fleahopper, *Pseudatomoscelis seriatus* (Reuter) (Hemiptera: Miridae), is an early-season pest of cotton, *Gossypium hirsutum* L. (Malvales: Malvaceae), that feeds on tender new growth, particularly the developing pre-floral reproductive buds known as squares. Using its piercing–sucking mouthparts, it penetrates individual plant cells, causing cell death [1]. High cotton fleahopper feeding pressure during early squaring can cause substantial square abscission, resulting in boll loss and reduced yield. Early square loss can also delay crop maturity if plants compensate by maturing later-developing bolls [2]. While cotton fleahopper infestations can lead to economic yield loss, the level of damage and the plant’s compensatory response depend on factors such as the timing of infestation, cultivar characteristics, and agronomic practices such as irrigation and fertility management [3,4]

The widespread adoption of transgenic cultivars targeting lepidopteran pests has significantly reduced the reliance on broad-spectrum insecticide application for managing cotton pests [5]. Since 1996, the deployment of plant-incorporated insecticidal Cry proteins derived from the soil bacterium *Bacillus thuringiensis* (Berliner) (Bt) has reduced the reliance on foliar insecticide application for heliothine (‘worm’) (Lepidoptera: Noctuidae) control in cotton [6]. This shift has heightened the economic importance of the cotton fleahopper in Texas and neighboring U.S. states and northeast Mexico, along with other plants bugs such as *Lygus* spp. that occur in this region and elsewhere [5,6]. To mitigate these effects, one or two foliar insecticide applications are typically used during the first three to four weeks of squaring, when cotton fleahopper is present and the plants are most susceptible to injury [4]. However, these applications are costly and can reduce populations of natural enemies [5].

Since the mid-1990s, Bt cottons expressing lepidopteran-active Cry toxins (e.g., Cry1Ac in first-generation Bollgard and Cry1Ac + Cry2Ab in pyramid products) have been highly effective against key bollworm pests. In combination with sterile insect release programs, this technology contributed to the eradication of the pink bollworm, *Pectinophora gossypiella*, from the southwest U.S. and northern Mexico [7]. However, field-evolved resistance to Cry1Ac and Cry2Ab has been documented in pink bollworm populations in India [8], and resistance in *Helicoverpa zea* populations in the U.S. has reduced the resilience and field performance of Cry-based traits [9]. These limitations highlight the need for transgenic technologies with novel modes of action and for combining Bt traits within an integrated pest management (IPM) framework.

This rise of hemipteran plant bugs and other piercing–sucking insects as important pests of cotton has justified the exploration of transgenic traits that confer protection against these pests to build on transgenic cultivars that control heliothines. Historically, the Cry proteins expressed in cotton cultivars have shown no efficacy against hemipteran pests, in association with binding affinity in the insect midgut or degradation by pre-ingestion enzymes [10]. However, the introduction of the Bt event Mpp51Aa2 (Cry51Aa2) into cotton (we henceforth use the common terminology, stating that these cultivars have the ThryvOn trait) has produced the first cultivars with efficacy against hemipterans and thysanopterans [10,11]. Similar to other Cry proteins, the Mpp51Aa2 pesticidal protein acts by targeting epithelial cells in the insect midgut, resulting in Bt crystalline inclusions and disruption of gut integrity [10].

Although behavioral effects of the ThryvOn trait on cotton fleahopper have been demonstrated, less is known about how this trait influences cotton fleahopper injury and plant responses under field conditions [12,13]. In this study, we evaluated early-season cotton fleahopper feeding on cultivars expressing the ThryvOn trait (Bayer CropScience, St. Louis, MO, USA) by assessing both direct effects, square loss and yield, and indirect effects related to plant maturity delay, measured by the open boll ratio. Because near-isogenic ThryvOn and non-ThryvOn cultivars were not available, the study focused on cultivar-level performance under field conditions, while recognizing that differences in genetic background and maturity class may influence the observed responses. The management premise was that cotton fleahopper populations can be reduced using cultivars with the ThryvOn trait alone or in combination with insecticide application, which results in yield protection and prevents delays in crop maturity.

## 2. Materials and Methods

### 2.1. Field Experimental Treatments and Design

Cotton fleahopper abundance and plant responses were assessed on four cotton cultivars differing in presence of the ThryvOn trait and maturity characteristics in a field experiment located in Corpus Christi, Texas. The cultivars were planted at a density of 98,800 seeds per hectare on a half-hectare field site in mid-April 2024 and 2025. The cultivars and maturity ratings were 24R6542 B3TXF (2024) (with ThryvOn trait and mid- to full-season maturity rating), 24R6522 B3TXF (2025) (with ThryvOn trait and early- to mid-season maturity rating), DP 2131 B3TXF (2024, 2025) (ThryvOn trait, mid/full-season), DP 2020 B3XF (2024, 2025) (no ThryvOn trait, early/mid-season), DP 2317 B3XF (2024) (no ThryvOn trait, early/mid-season), and DP 2239 B3XF (2025) (no ThryvOn trait, mid/full-season). Because near-isogenic lines were not available, comparisons between ThryvOn and non-ThryvOn cultivars necessarily include differences in genetic background and maturity class, which limits attribution of observed responses solely to the Mpp51Aa2 (ThryvOn) trait. In addition, the cultivar sets evaluated differed between 2024 and 2025, reflecting commercial availability and maturity class representation (see Supplemental Table S1). Consequently, treatment effects were evaluated primarily within years, and year-to-year comparisons are interpreted cautiously with respect to trait consistency.

The cotton fleahopper populations were naturally occurring. Planting was delayed relative to regional norms to increase the likelihood that populations would exceed the established economic threshold. Cotton fleahopper abundance was assessed using a beat bucket sampling method, with a threshold of 0.25 cotton fleahoppers per plant during the first four weeks of squaring [14]. With this strategy, cotton fleahopper populations rose to the economic threshold midway through (2024) and late (2025) during this period, as observed in plots without application of foliar insecticide treatment (see below). Weed management practices were implemented uniformly in the plots and consisted of two herbicide applications: a pre-emergence application of S-metolachlor (Dual Magnum, Syngenta, Greensboro, NC, USA) and a post-emergence application of glyphosate (Roundup PowerMax, Bayer CropScience, St. Louis, MO, USA).

The experiment followed a split-plot design with six and five replications in 2024 and 2025, respectively. Each whole plot measured 12 by 18 m, with 12 rows per plot. The four cotton cultivars were the main plots. Each main plot was divided into two subplots: one experiencing natural infestations of cotton fleahopper and one protected from cotton fleahopper by foliar application of insecticide. The insecticide flupyradifurone (Sivanto Prime, Bayer CropScience, St. Louis, MO, USA) was applied twice in the protected subplots during the first four weeks of squaring, beginning in the last week of May. Each application delivered 511.6 mL of product (102.3 g active ingredient) per hectare using a Spra-Coupe sprayer (Duluth, GA, USA) calibrated to apply 186.9 L of water per hectare.

### 2.2. Experimental Measurements: Insect Monitoring and Plant Responses

Cotton fleahopper populations were monitored, starting with the first appearance of pinhead-sized cotton squares for four weeks using the beat bucket method [14]. Twenty plants were sampled from the center of the 12-row plots to minimize insect disturbance and movement between plots. Briefly, the foliage of each plant was bent quickly and shaken vigorously in an 18.9 L (five-gallon) bucket to dislodge insects. Nymph and adult counts were used to estimate densities on a per plant basis each week. A representative week of each year showing treatment effects is reported here.

For plant response measurements, each cotton plant was divided into three sections: the bottom, middle, and top thirds of the plant [15]. The bottom and middle sections each consisted of five branches starting from the plant base, while any remaining branches were classified as the top section. The bottom section contained the oldest growth, bearing the earliest-formed squares and bolls of the first and second positions from the main stem. These parts were vulnerable to cotton fleahopper that infested the crop during early squaring but may have escaped injury from cotton fleahopper arriving later. In contrast, the top section comprised the latest-maturing bolls that typically contribute less to yield. Dividing the cotton plant in this manner enabled the evaluation of plant productivity relative to the timing of cotton fleahopper activity. For example, cotton fleahopper arriving early in square initiation threatens the major yield-contributing bolls on the bottom third of the plant and other sections if populations are left unmanaged. In contrast, cotton fleahopper first arriving in the third or fourth week of squaring shifts risk to squares and bolls on the middle and top sections, while the risk to the bottom section is lower.

Plant measurements included the ratio of abscised squares to total squares (in 2025 only due to weather and logistic challenges), the ratio of open bolls to total bolls (as a measure of delayed maturity), and seed cotton weight taken by hand and machine harvesting. Lint weight was measured in 2024 but not in 2025 because it paralleled seed cotton yield in 2024, and significant differences in seed cotton weight across cultivars were not detected in 2025. Therefore, seed cotton yield for both years is presented here. One week after the second flupyradifurone application in 2025, the number of squares that appeared healthy (uninjured) and that experienced some form of wounding (injured), including response to cotton fleahopper feeding, was recorded at the first two fruiting positions from the main stem on each branch. Ten consecutive plants per subplot were inspected, with each plant section assessed separately for uninjured and injured squares. One week before harvest, timed according to the harvest schedule of cultivars with full-season maturity ratings (at ca. 140 days post-emergence), the numbers of open and green bolls on 10 consecutive plants per subplot were recorded. This assessment was used to calculate the open boll ratio separately for each section of the cotton plant. Seed cotton weights were estimated on a whole-plant basis using machine harvesting and by plant section through hand picking. Machine harvesting was carried out with a JD9930 cotton picker (John Deere, Moline, IL, USA), which harvested whole plants from a 10.7 m section on a single inner row not previously used for insect and plant measurements. Hand harvesting was performed by collecting open bolls by hand from the bottom, middle, and top sections along a uniform row, covering a 4.6 to 6.1 m section within each subplot. The seed cotton weight from each subplot was converted to kilograms per hectare (kg/ha). See Supplemental Figures S1–S4 for photos of square injury, sampling method, and plant assessment

### 2.3. Data Analysis

All measurements were analyzed using an analysis of variance (ANOVA) appropriate for a split-plot design [16]. Cotton cultivars (Cultivar), differing in the presence of the ThryvOn trait and maturity rating, were treated as a fixed main-plot factor, while cotton fleahopper pressure (Fleahopper; sprayed vs. unsprayed), manipulated through insecticide applications, was treated as a fixed subplot factor. Replication was included as a blocking factor. The main-plot effect of cultivar was tested using the replication × cultivar interaction as the error term, whereas the subplot effect of cotton fleahopper pressure and the cultivar × cotton fleahopper interaction were tested using the residual error term. The open boll ratio measurement was used as a covariate in the ANOVAs for the other plant measurements, given the difference in maturity ratings among the cultivars and its potential influence on plant responses to cotton fleahopper injury. Data were analyzed using SAS software (Version 9.4; SAS Institute Inc., Cary, NC, USA) with a generalized linear model (PROC GLM). Count data were transformed using the square root of (count + 0.5), while the ratio data (squares and bolls) were transformed using the arcsine square root transformation. The transformations were standard and applied to satisfy statistical requirements for normality and homogeneity of variance for the ANOVA [17]. All statistical tests were performed at a significance level of α = 0.05. Data from the three plant sections were analyzed separately.

## 3. Results

### 3.1. Cotton Fleahopper Abundance

**Figure 1:**
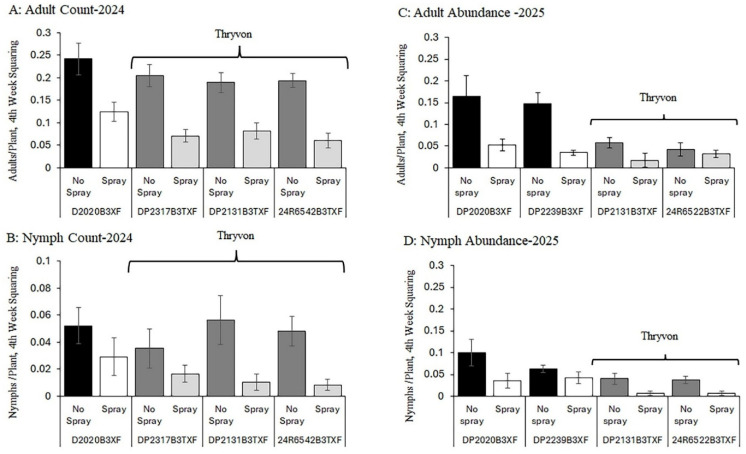
Abundance of cotton fleahopper per plant during the fourth week of squaring in 2024 ((**A**) adults and (**B**) nymphs) and 2025 ((**C**) adults and (**D**) nymphs) on cotton cultivars differing in the presence of the ThryvOn trait and maturity (early/mid-season and mid/full-season maturity [Supplemental Table S1]). Subplots of cultivars were not sprayed or sprayed with insecticide twice to produce relatively high (no spray) and low (spray) cotton fleahopper pressure. Each bar presents the mean ± SE (standard error [line]).

In 2024, no interaction was detected between cotton cultivar (main plot) and cotton fleahopper pressure (split plot) for either adults or nymphs (Cultivar × Fleahopper interaction: *p* > 0.60); therefore, we focused on inspecting the two factors separately. As expected, the abundance of cotton fleahopper, both adults and nymphs, was significantly higher in subplots with natural populations of cotton fleahopper than in subplots sprayed twice with insecticide during the first four weeks of squaring (Fleahopper main effect, F > 12.0, df = 1, 20, *p* < 0.005). This pattern was evident from the second through the fourth week of squaring, when populations exceeded the economic threshold of 0.25 cotton fleahopper per plant (Figure 1A,B).

Regarding the ThryvOn trait comparison, adult cotton fleahopper abundance differed among cultivars during the fourth week of squaring (Cultivar main effect, F = 3.48, df = 3, 20, *p* = 0.04 [Figure 1A]), with cultivars expressing the ThryvOn trait having fewer adults than cultivars without the trait. In contrast, nymph abundance did not differ significantly among cultivars (Cultivar, *p* = 0.30 [Figure 1B]). Although nymph populations were low and variable, this pattern was consistent with the interpretation that cultivars expressing the ThryvOn trait were less susceptible to initial cotton fleahopper infestation (i.e., early-season adult colonization), whereas reproduction was not affected and appeared to offset the small reductions observed in adult abundance.

In 2025, similar patterns were observed; however, overall pest pressure was lower than in 2024, with cotton fleahopper populations only approaching the economic threshold during the fourth week of squaring (Figure 1C,D). At these lower population levels, differences among cultivars were more clearly resolved, with fewer adult cotton fleahoppers observed on cultivars expressing the ThryvOn trait (DP 2131 B3TXF and 24R6522B3TXF) compared with non-ThryvOn cultivars (cultivar effect: F = 22.26, df = 3, 16, *p* < 0.0001 [Figure 1C]). Differences in adult abundance among cultivars were sufficient to result in fewer cotton fleahopper nymphs on ThryvOn cultivars (cultivar effect: F = 4.03; df = 3, 16, *p* = 0.03 [Figure 1D]).

Across both years, the ThryvOn cultivars exhibited modest reductions in adult cotton fleahopper abundance compared to the non-ThryvOn cultivars. In 2024, these reductions averaged ~10–25%, corresponding to mean differences of approximately 0.05–0.12 adults per plant during the fourth week of squaring. In 2025, when pest pressure was lower, adult populations were reduced by ~15–30% on ThryvOn cultivars, with mean differences of ~0.04–0.10 adults per plant at population levels approaching the economic threshold.

### 3.2. Square Abscission

**Figure 2:**
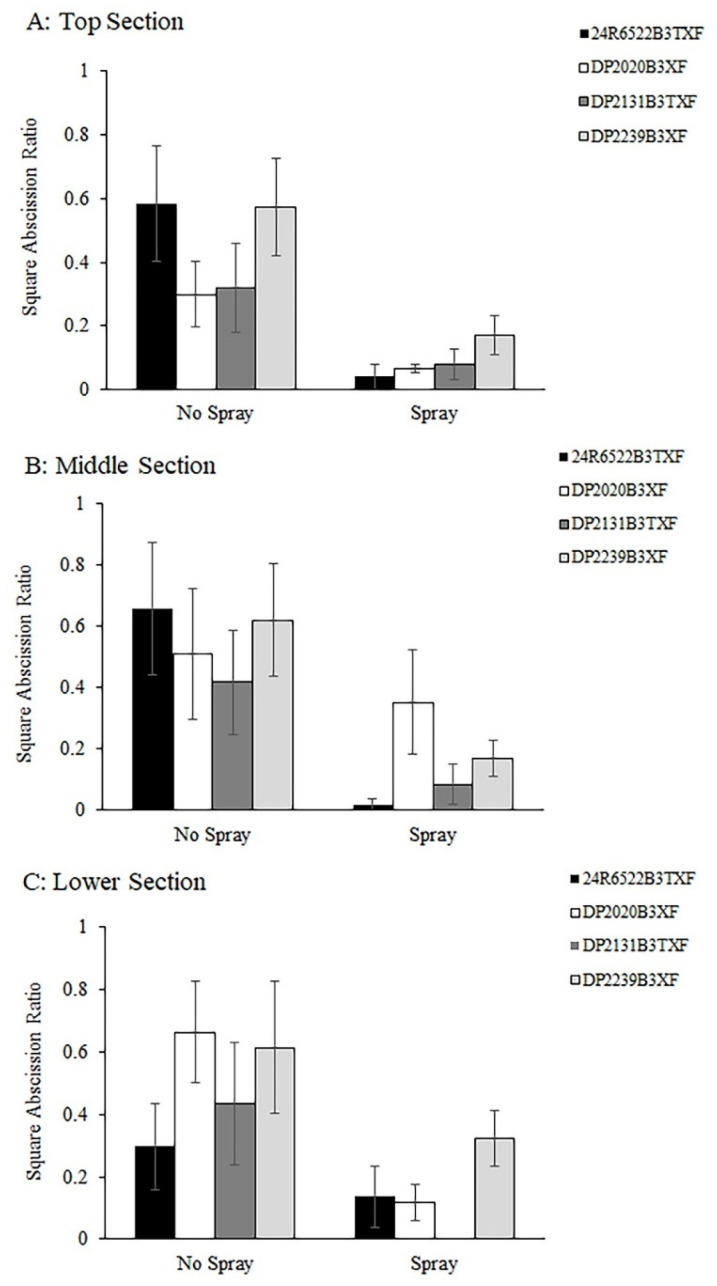
Square abscission ratios in response to cotton fleahopper pressure across three plant sections per plant in 2025 at the top (**A**), middle (**B**), and lower (**C**) sections of four cultivars differing in the presence of the ThryvOn trait and maturity (early/mid-season and mid/full-season maturity [Supplemental Table S1]). Subplots of cultivars were not sprayed or sprayed with insecticide twice to produce relatively high (no spray) and low (spray) cotton fleahopper pressure. Each bar presents the mean ± SE (standard error [line]).

Square abscission data were not collected in 2024 due to weather and logistical constraints. Consequently, square abscission was evaluated only in 2025 with ANOVA, including the open boll ratio as a covariate. No interaction was detected between cotton cultivar and cotton fleahopper pressure for any plant section (top, middle, or lower; *p* ≥ 0.20); therefore, the main effects of these factors were evaluated separately. Square abscission was significantly higher in subplots with natural cotton fleahopper infestations (i.e., without foliar insecticide applications), where populations exceeded the economic threshold during the fourth week of squaring, than in insecticide-protected subplots across three plant sections (Figure 2). This effect was observed in the top section (Fleahopper: F = 26.50, df = 1, 16, *p* < 0.0001 [Figure 2A]), middle section (F = 12.15, df = 1, 16, *p* = 0.002 [Figure 2B]), and lower section (F = 15.50, df = 1, 16, *p* = 0.0012 [Figure 2C]). No significant differences in square abscission were detected among the four cotton cultivars differing in the presence of the ThryvOn trait for any of the three plant sections (top, middle, and lower sections, cultivar, *p* > 0.08 [Figure 2A–C]).

### 3.3. Open Boll Ratio

**Figure 3:**
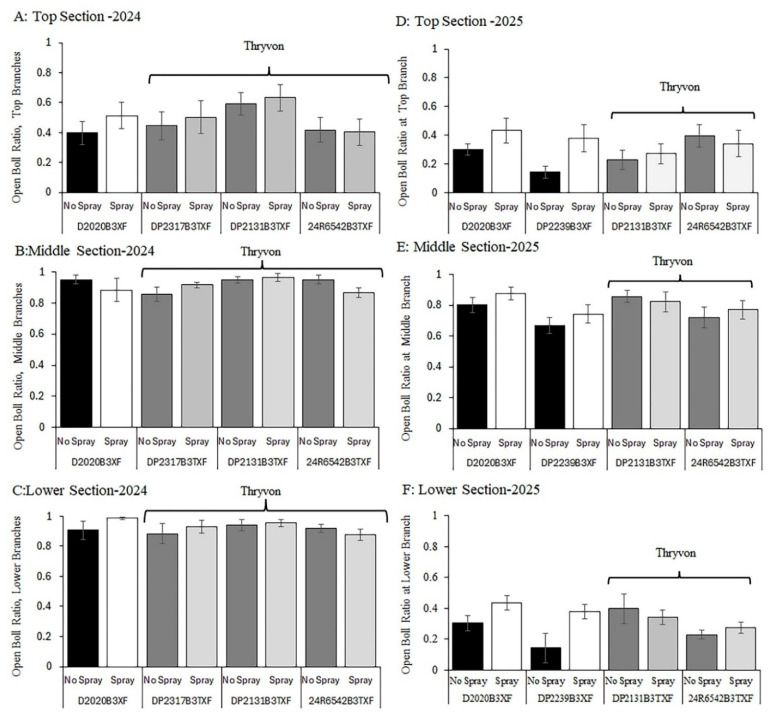
The open boll ratio in response to cotton fleahopper pressure was assessed one week before harvest in the top (2024 (**A**); 2025 (**D**)), middle (2024 (**B**); 2025 (**E**), and lower (2024 (**C**); 2025 (**F**)) sections of four cotton cultivars differing in the presence of the ThryvOn trait and maturity (early/mid-season and mid/full-season maturity [Supplemental Table S1]). Subplots of cultivars were unsprayed or sprayed with insecticide twice to produce relatively high (no spray) and low (spray) cotton fleahopper pressure. Each bar presents the mean ± SE (standard error [line]).

In 2024, no interaction between cultivar and cotton fleahopper pressure was detected for open boll ratio across the three plant sections (*p* > 0.36); therefore, the two factors were considered separately. No significant differences in open boll ratio were observed among the four cultivars (Cultivar effect: *p* > 0.19) or between subplots with natural cotton fleahopper infestations and those protected with insecticide applications (Fleahopper effect: *p* > 0.13) at the top (Figure 3A), middle (Figure 3B), or lower (Figure 3C) plant sections. In 2025, a similar pattern was observed, with no significant cultivar × cotton fleahopper interactions (*p* > 0.17) and no significant main effects of cultivar (*p* > 0.46) or cotton fleahopper pressure (*p* > 0.50) across the three plant sections (Figure 3D–F).

### 3.4. Seed Cotton Weight by Hand Harvest of Three Plant Sections

**Figure 4:**
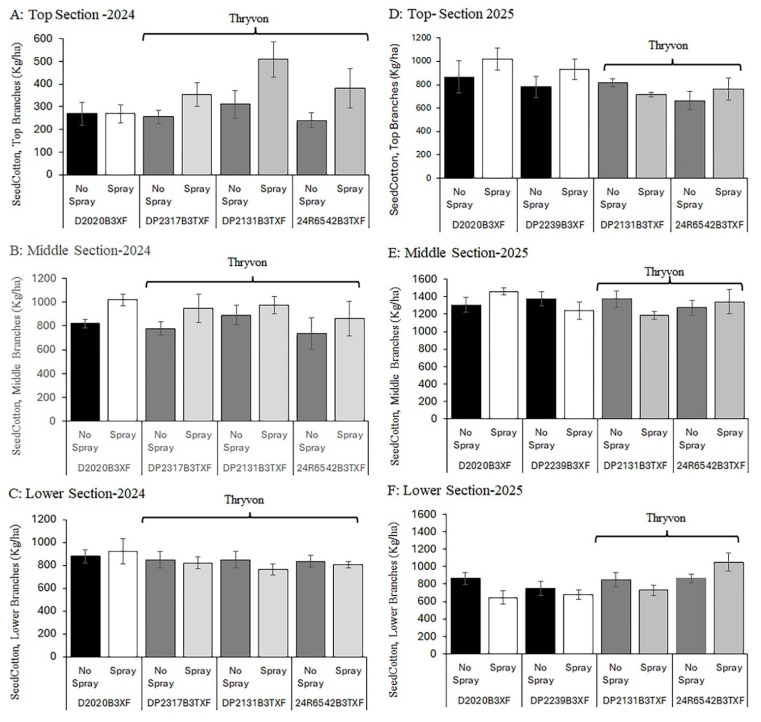
Seed cotton weight was taken by hand harvesting and was assessed in response to cotton fleahopper pressure in the top (2024 (**A**); 2025 (**D**)), middle (2024 (**B**); 2025 (**E**)), and lower (2024 (**C**); 2025 (**F**)) sections of cotton plants for four cultivars differing in the presence of the ThryvOn trait and maturity (early/mid-season and mid/full-season maturity [Supplemental Table S1]). Subplots of cultivars were not sprayed or sprayed with insecticide twice to produce relatively high (no spray) and low (spray) cotton fleahopper pressure. Each bar presents the mean ± SE (standard error [line]).

Seed cotton weight estimated by hand and machine harvesting was evaluated by ANOVA that included the open boll ratio as a covariate. In 2024, no cultivar × cotton fleahopper interaction was detected for seed cotton weight across the three plant sections (*p* > 0.30); therefore, the two factors were evaluated separately. Seed cotton weight was significantly higher in insecticide-protected subplots, which had lower cotton fleahopper densities, than in subplots with natural cotton fleahopper infestations that reached the economic threshold during the second week of squaring at the top (Fleahopper effect, F = 7.78, df = 1, 20, *p* = 0.011 [Figure 4A]) and middle plant sections (F = 18.87, df = 1, 20, *p* = 0.0003 [Figure 4B]) but not in the lower section (*p* = 0.49 [Figure 4C]). Regarding the ThryvOn trait comparison, no significant differences in seed cotton weight were detected among the four cultivars in any of the three plant sections (Cultivar effect, *p* > 0.15 [Figure 4A–C]).

In 2025, seed cotton weight exhibited greater variability than in 2024. One significant cultivar × cotton fleahopper interaction was detected in the middle plant section (F = 5.18, df = 3, 16, *p* = 0.011), but no patterns of additivity or synergy of using insecticide protection and cultivars with the ThryvOn trait were discernable at the cotton fleahopper pressure experienced here. Looking at the main effects. differences among cultivars were observed only in the lower plant section, where seed cotton weight was higher for the noncommercial cultivar with the ThryvOn trait and rated as maturing relatively early (24R6522 B3TXF; cultivar effect: F = 4.51, df = 3, 16, *p* = 0.025 [Figure 4F]). Unexpectedly, no significant differences in seed cotton weight were detected between insecticide-protected and untreated subplots across the three plant sections (*p* > 0.10 [Figure 4D–F]). As noted above, cotton fleahopper populations did not approach the economic threshold until the fourth week of squaring. Overall, in 2024, insecticide protection resulted in an increase in seed cotton yield of approximately 250–400 kg ha^−1^ in the top and middle plant sections, whereas differences among cultivars within sections were generally <150 kg ha^−1^. In 2025, yield differences between insecticide-protected and unsprayed subplots were <200 kg ha^−1^ across plant sections, consistent with cotton fleahopper populations remaining near or below the economic threshold.

### 3.5. Seed Cotton Weight from Machine Harvesting

**Figure 5:**
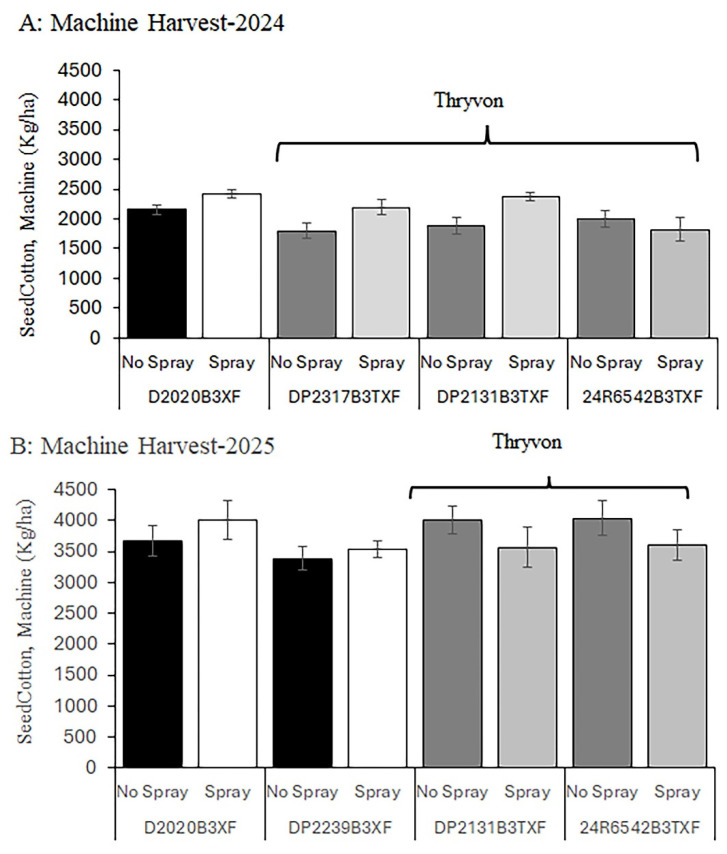
Seed cotton weight was taken by machine harvesting and assessed in response to cotton fleahopper pressure on four cultivars differing in the presence of the ThryvOn trait and maturity (early/mid-season and mid/full-season maturity [Supplemental Table S1]). Subplots of cultivars were not sprayed or sprayed with insecticide twice to produce relatively high (no spray) and low (spray) cotton fleahopper pressure. Each bar presents the mean ± SE (standard error [line]).

In 2024, a significant cultivar × cotton fleahopper interaction was detected for machine-harvested seed cotton weight (F = 3.05, df = 3, 20, *p* = 0.05). One cultivar expressing the ThryvOn trait (24R6542 B3TXF) exhibited relatively high yield under natural cotton fleahopper pressure, whereas the remaining cultivars, with and without the ThryvOn trait, produced higher yields in insecticide-protected subplots (Figure 5A). When main effects were examined separately, seed cotton weight was significantly higher in insecticide-protected subplots than in untreated subplots (Fleahopper main effect: F = 8.56, df = 1, 20, *p* = 0.009). No differences in machine-harvested seed cotton weight were detected among the four cotton cultivars (Cultivar, *p* = 0.07 [Figure 5A]). In 2025, no cultivar × fleahopper interaction was detected for machine-harvested seed cotton weight (*p* = 0.15), and no main effects of cultivar (Cultivar, *p* = 0.46) or cotton fleahopper pressure (Fleahopper, *p* = 0.57) were observed (Figure 5B). Overall, in 2024, flupyradifurone applications increased machine-harvested seed cotton yield by approximately 350–450 kg ha^−1^ across cultivars, whereas differences among cultivar means were generally <200 kg ha^−1^. In 2025, yield differences among treatments were small (<150 kg ha^−1^) and not statistically detectable.

## 4. Discussion

Across both years, cotton fleahopper abundance was reduced by two flupyradifurone applications during the first four weeks of squaring. In contrast, cultivars expressing the ThryvOn trait showed only small and variable effects on adult and nymph cotton fleahoppers (reductions of ~10–30%). These insecticide and ThryvOn cultivar effects occurred at natural population levels that peaked near the economic threshold (midway through this period in 2024 and later in 2025) on cultivars without the ThryOn trait and without insecticide protection. Early-season flupyradifurone application was the primary factor influencing cotton fleahopper population control, resulting in yield protection in 2024. In contrast, cultivars with and without the ThryvOn trait had similar yields. In addition, the open boll ratio, as an indicator of maturity, was not significantly affected by the cultivars or cotton fleahopper pressure in either year, suggesting that early-season cotton fleahopper injury did not result in observable delays in boll development under the cotton fleahopper population levels seen in this study.

Overall, these findings indicate that although cultivars with the ThryvOn trait influenced cotton fleahopper abundance, early-season flupyradifurone applications were overwhelmingly responsible for the effect on cotton fleahopper and yield protection. This pattern was similar across maturity ratings of the cultivars used. These findings indicate that cultivars with the ThryvOn trait may supplement to a modest degree, but not replace nor substantially increase, the benefits in cotton fleahopper control provided by early-season flupyradifurone applications. In our field setting, cotton fleahopper had ample opportunity to be exposed to the Bt protein associated with the ThryvOn trait through its feeding on squares. Gowda et al. [11] documented that the protein was expressed at higher levels in squares than in bolls and leaves. There are possible protein sensitivity differences across hemipteran plant bug species. In a caged study under greenhouse conditions, a 1.7-fold reduction in cotton fleahopper was seen in the second generation when placed on cotton with this trait (compared with placement on cotton without this trait), while a 15-fold reduction was seen using *Lygus lineolaris* [10]. In addition, Akbar et al. [18] collected two *Lygus* species on a cotton cultivar expressing Cry51Aa2.834_16 (MON 88,702 event) and its non-transgenic near-isoline cultivar DP393 with and without the use of *Lygus*-active insecticide sprays. Insects were reduced consistently with synergism between insecticides and the Bt trait, but only in locations with naturally high *Lygus* pressure.

For our field application where cotton fleahopper is a key pest, these findings are best interpreted within the broader context of Bt cotton technologies. While the ThryvOn trait represents a novel extension of Bt-based management of piercing–sucking pests [10,11,19], its effects on cotton fleahopper abundance were modest and did not translate into consistent reductions in square abscission or increased yield protection. This result is in contrast to the strong and consistent benefits of Bt traits targeting selected lepidopteran pests under field conditions [5]. Insecticide use for hemipteran control can be minimized by the use of established economic thresholds and sampling techniques for cotton fleahopper and other hemipterans [14,20]. Reduced use also minimizes effects on non-target beneficial insects that have been observed when using novel insecticides like flupyradifurone [21]. In the southern U.S., where this study was conducted, there are no requirements to plant non-Bt cotton cultivars as refuge because natural refuges (wild hosts or other cultivated crops that serve as sources of susceptible insects) are considered sufficient to minimize risk to insect resistance development to the Bt transgenes incorporated into cotton [22].

Interpretation of the ThryvOn-related effects should be made with some caution, as the cultivars compared were not near-isogenic and differed in both maturity class and genetic background. Variation in measurements (i.e., square abscission, adult and nymphal densities, plant maturity delays, yield) across the cotton cultivar and insecticide treatments applied in this study also may be associated with the different insect population levels and environmental conditions, particularly plant water stress, between years. Last, because the cultivar sets differed between 2024 and 2025, year-to-year comparisons reflect qualitative trends rather than quantitative assessments of trait consistency. Despite these limitations, the pattern of modest and variable ThryvOn effects contrasted with strong insecticide control. The results support the conclusion that early-season flupyradifurone applications were the primary factor influencing cotton fleahopper suppression and yield protection under the conditions evaluated. With the advisable continued use of early-season insecticides like flupyradifurone for control of cotton fleahopper, along with use of Bt cottons for at least heliothine control, insecticide resistance management (IRM) and integrated pest management (IPM) remain considerations in the combined use of insecticides and Bt cottons. Bt-cottons with the ThryvOn trait, although lacking substantial cotton fleahopper control in this study, do provide protection from other pest species, particularly thrips spp. (Thysanoptera: Thripidae) [10,19] and, to a lesser extent, *Lygus* spp. [10].

For future field experiments with cotton fleahopper and other hemipteran pests that may respond to the ThryvOn trait, we suggest increasing standardization and measurement of key insect and environmental conditions. For cotton fleahopper population levels, late plantings to increase the likelihood of natural infestations above the economic threshold during early squaring can be further augmented by surveying weedy hosts of the insect (e.g., wooly croton, *Croton capitatus* Michx [4]), collecting infested material, and distributing the material in the experimental plots. Regarding environmental conditions, plant water stress is a key factor affecting cotton’s response to cotton fleahopper feeding, including square abscission [4]. Because our Corpus Christi site commonly experiences droughty conditions and the region’s highly textured soils can maintain differences in soil moisture across small plots, we often are in a good position to manipulate plant water stress experimentally using drip irrigation [4]. Alongside controlled manipulation, environmental conditions should be monitored when insect data are collected on transgenic and non-transgenic cultivars planted in multiple locations that vary in temperature, rainfall, and humidity [18]. The increased manipulation of cotton fleahopper pressure and plant water stress aids year-to-year standardization and allows for further consideration of any synergistic effects of combining the ThryvOn trait with insecticides. Continued study at higher cotton fleahopper population levels during early squaring and under plant water stress conditions would be useful to confirm the observations made in this study, conducted under natural insect population levels rising to but not substantially exceeded the economic threshold and crop-favorable conditions.

## Data Availability

The original contributions presented in this study are included in the article and its Supplementary Material. Further inquiries may be directed to the corresponding author.

## Supplementary Materials

The following supporting information can be downloaded at https://www.mdpi.com/article/10.3390/insects17030233/s1, Figure S1: Cotton square showing feeding injury caused by adult cotton fleahopper (*Pseudatomoscelis seriatus*). Photo credits: MA (left) and R. Leonard (right); Figure S2: Beat-bucket sampling method used to quantify cotton fleahopper adults and nymphs during the squaring period. Photo credit: MA; Figure S3: Open boll ratio assessment showing bottom, middle, and top plant sections evaluated prior to harvest. Photo credit: MA; Figure S4: Schematic representation of cotton plant division into bottom, middle, and top sections for yield and fruit retention assessments. Photo credit: MA; Table S1: Commercial upland cotton (*Gossypium hirsutum* L.) cultivars evaluated in field experiments (Corpus Christi, Texas, USA; 2024–2025), including maturity class and presence of the Mpp51Aa2 (ThryvOn) trait; Table S2: Mean (±SE) number of adult and nymph cotton fleahoppers per plant for each cultivar under sprayed and no-spray treatments, 2024; Table S3: Mean (±SE) number of adult and nymph cotton fleahoppers per plant for each cultivar under sprayed and no-spray treatments, 2025; Table S4: Mean (±SE) square abscission for each cultivar under sprayed and no-spray treatments, 2025; Table S5: Mean (±SE) open boll ratio for each cultivar under sprayed and no-spray treatments, 2024; Table S6: Mean (±SE) open boll ratio for each cultivar under sprayed and no-spray treatments, 2025; Table S7: Mean (±SE) seed cotton weight (hand harvest) for each cultivar under sprayed and no-spray treatments, 2024; Table S8: Mean (±SE) seed cotton weight (hand harvest) for each cultivar under sprayed and no-spray treatments, 2025; Table S9: Mean (±SE) seed cotton weight (machine harvest) for each cultivar under sprayed and no-spray treatments, 2024–2025.
