# Supplementary material for: Effect of Transgenic Cotton with Bt Event Mpp51Aa2 on Cotton Fleahopper (Pseudatomoscelis seriatus) During Early Cotton Growth and Resulting Plant Injury
Source: Insects. 2026 Feb 24;17(3):233. doi: 10.3390/insects17030233 (PMC13026286; doi:10.3390/insects17030233)
Supplement: Supplementary file 1 [file insects-17-00233-s001.zip › insects-4090049-supplementary.pdf]

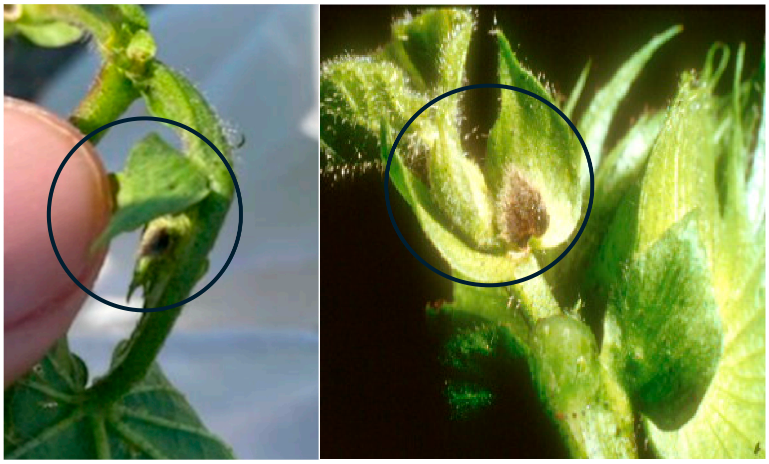

Supplementary Figure S1 A cotton square showing feeding injury caused by adult cotton fleahopper (*Pseudatomoscelis seriatus*). Left photo credit MA. Right photo courtesy of R. Leonard.

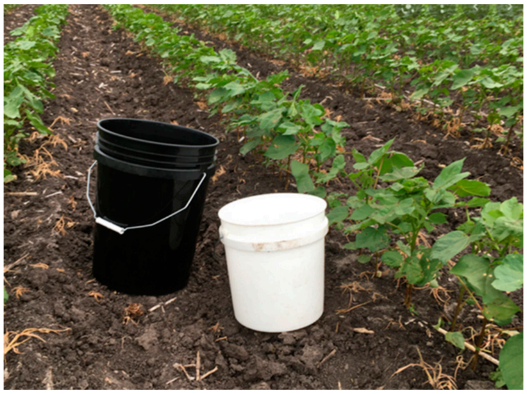

Figure S2 Beat-bucket sampling method used to quantify cotton fleahopper adults and nymphs during the first four weeks of squaring. Plants were struck over a beat bucket to remove cotton fleahopper for standardized field counts. Photo credit MA.

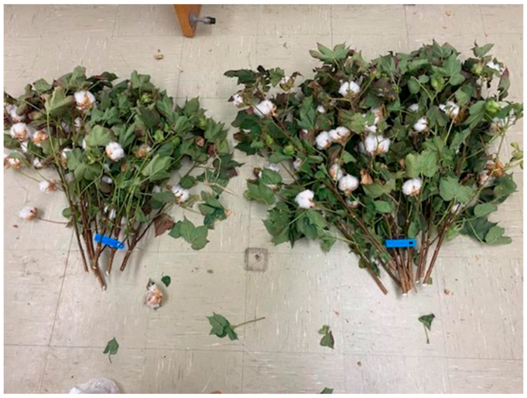

Figure S3 Open boll ratio assessment conducted one week prior to harvest, showing the bottom, middle, and top sections of cotton plants. Each section consisted of four to five fruiting branches, with ten plants evaluated per plot. Photo credit MA.

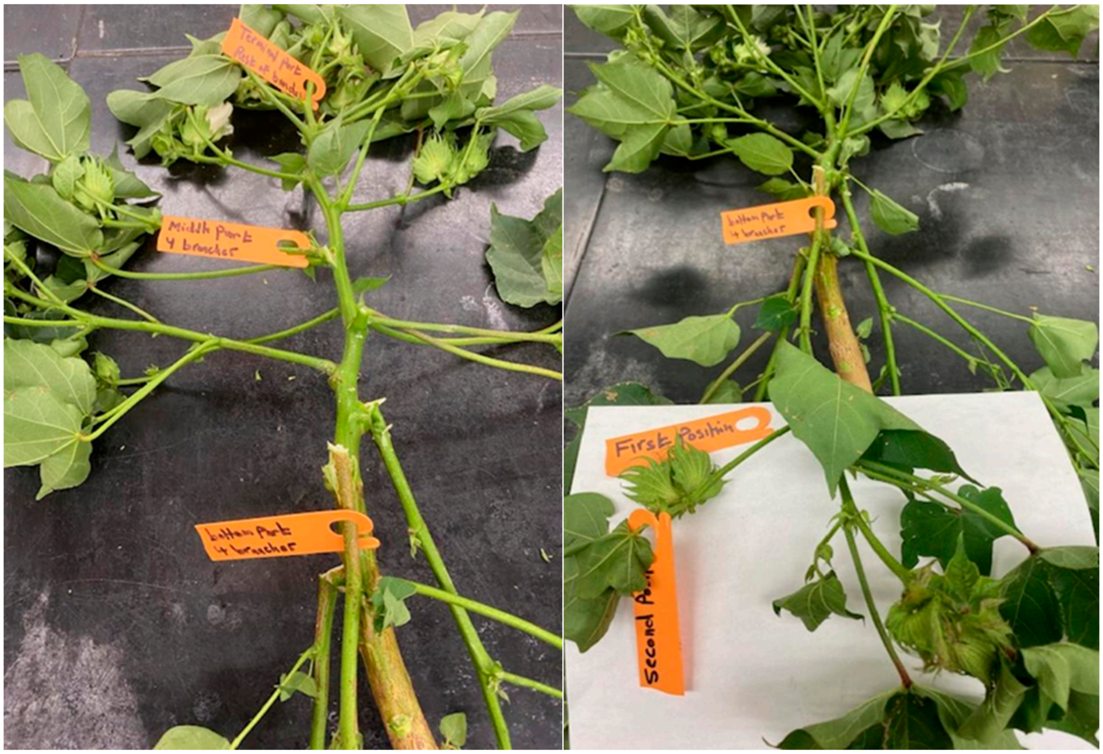

Figure S4 Representation image of cotton plant dividing used for yield and fruit retention assessments. Plants were divided into bottom, middle, and top sections, with each section comprising four to five fruiting branches. Photo credit MA.

Supplementary Table S1. Commercially available upland cotton (*Gossypium hirsutum* L.) cultivars evaluated in field experiments conducted in Corpus Christi, Texas, USA, in 2024 and 2025, including maturity classification and the presence or absence of the Mpp51Aa2 (Cry51Aa2; ThryvOn) trait**.**

Year	Cultivar	ThryvOn Trait	Maturity Classification
2024	DP 2020 B3XF	No	Early to mid-season
2024	DP 2317 B3TXF	Yes	Early to mid-season
2024	DP 2131 B3TXF	Yes	Mid to full-season
2024	24R6542 B3TXF	Yes	Mid to full-season
2025	DP 2020 B3XF	No	Early to mid-season
2025	DP 2239 B3XF	No	Mid to full-season
2025	DP 2131 B3TXF	Yes	Mid to full-season
2025	24R6522 B3TXF	Yes	Early to mid-season

Supplementary Table S2. Mean (± SE) number of adult and nymph cotton fleahoppers per plant for each cotton cultivar under sprayed and no spray treatments during the 2024 growing season. Means were calculated across sampling dates during the squaring period.

Adult Abundance 2024			
Cultivar	Treatment	Mean_ Adults/Plant	SE_Nymph/Plant
D2020B3XF	No Spray	0.241666667	0.035009919
	Spray	0.125	0.021408721
DP2317B3TXF	No Spray	0.204166667	0.024509069
	Spray	0.070833333	0.01431297
DP2131B3TXF	No Spray	0.189583333	0.022224392
	Spray	0.08125	0.017897276
24R6542B3TXF	No Spray	0.19375	0.015394128
	Spray	0.060416667	0.016588358

Nymph Abundance 2024			
Cultivar	Treatment	Mean_ Adults/Plant	SE_Nymph/Plant
D2020B3XF	No Spray	0.052083333	0.013469358
	Spray	0.029166667	0.013944334
DP2317B3TXF	No Spray	0.035416667	0.014583333
	Spray	0.016666667	0.006180165
DP2131B3TXF	No Spray	0.05625	0.017897276
	Spray	0.010416667	0.005965759
24R6542B3TXF	No Spray	0.047916667	0.01090521
	Spray	0.008333333	0.004166667

Supplementary Table S3. Mean (± SE) numbers of adult and nymph cotton fleahoppers abundance per plant for each cotton cultivar under sprayed and no spray treatments during the growing season of 2025. Means were calculated across sampling dates during the squaring period

Adult Abundance 2025			
**cultivar**	**Treatment**	Mean_ Adults/Plant	SE_Nymph/Plant
DP2020B3XF	No spray	0.165	0.046670387
	Spray	0.0525	0.013919411
DP2239B3XF	No spray	0.1475	0.026040833
	Spray	0.035	0.006123724
DP2131B3TXF	No spray	0.0575	0.012247449
	Spray	0.0175	0.015612495
24R6522B3TXF	No spray	0.0425	0.015612495
	Spray	0.0324	0.008477912

Nymph Abundance 2025			
**cultivar**	**Treatment**	Mean_ Adults/Plant	SE_Nymph/Plant
DP2020B3XF	No spray	0.1	0.029843341
	Spray	0.035	0.016955825
DP2239B3XF	No spray	0.0625	0.008838835
	Spray	0.0425	0.014031215
DP2131B3TXF	No spray	0.04	0.0121192
	Spray	0.0075	0.005
24R6522B3TXF	No spray	0.0375	0.008838835
	Spray	0.0075	0.005

Supplementary Table S4. Mean (± SE) of square abscission for each cotton cultivar under sprayed and no spray treatments during the 2025 growing season. Means were calculated across sampling dates during the squaring period.

Obs	Branch	cultivar	Treatment	Mean Abscission	SE_Abscission
1	Lower	24R6522B3TXF	No Spray	0.296666667	0.138483854
2	Lower		spray	0.136363636	0.097489139
3	Lower	DP2020B3XF	No Spray	0.66372549	0.162221577
4	Lower		spray	0.118333333	0.056297819
5	Lower	DP2131B3TXF	No Spray	0.434545455	0.197513469
6	Lower		spray	0	0
7	Lower	DP2239B3XF	No Spray	0.614285714	0.211409266
8	Lower		spray	0.323443223	0.089846973

Obs	Branch	cultivar	Treatment	Mean Abscission	SE_Abscission
1	Middle		No Spray	0.657142857	0.214761377
2	Middle	24R6522B3TXF	spray	0.018181818	0.018181818
3	Middle		No Spray	0.51	0.212367606
4	Middle	DP2020B3XF	spray	0.352727273	0.171777292
5	Middle		No Spray	0.416666667	0.170782513
6	Middle	DP2131B3TXF	spray	0.084848485	0.064567502
7	Middle		No Spray	0.62	0.18547237
8	Middle	DP2239B3XF	spray	0.170539986	0.059434038

Obs	Branch	cultivar	Treatment	Mean_Abscission	SE_Abscission
1	Top		No Spray	0.584344012	0.181148319
2	Top	24R6522B3TXF	spray	0.04	0.04
3	Top		No Spray	0.29928299	0.102726648
4	Top	DP2020B3XF	spray	0.065608466	0.011931188
5	Top		No Spray	0.319659091	0.141276053
6	Top	DP2131B3TXF	spray	0.08	0.048989795
7	Top		No Spray	0.5725	0.153927743
8	Top	DP2239B3XF	spray	0.170500114	0.059828743

Supplementary Table S5. Mean (± SE) of open boll ratio for each cotton cultivar under sprayed and no spray treatments during the 2024 growing season. Means were calculated across sampling dates during the squaring period.

Top			
Cultivar	Treatment	Mean Openboll Ratio	SE Open boll Ratio
D2020B3XF	No Spray	0.396725896	0.075981989
	Spray	0.513309679	0.086897206
DP2317B3TXF	No Spray	0.445258189	0.094224389
	Spray	0.501389014	0.109802451
DP2131B3TXF	No Spray	0.592455723	0.075562717
	Spray	0.631901189	0.087128651
24R6542B3TXF	No Spray	0.417018929	0.084546014
	Spray	0.402242735	0.08671283

Middle			
Cultivar	Treatment	Mean Openboll Ratio	SE Open boll Ratio
D2020B3XF	No Spray	0.950505051	0.028680902
	Spray	0.883976318	0.075167736
DP2317B3TXF	No Spray	0.857189755	0.046059216
	Spray	0.91513714	0.016842252
DP2131B3TXF	No Spray	0.946893233	0.021140896
	Spray	0.965116279	0.026685139
24R6542B3TXF	No Spray	0.950697105	0.029552921
	Spray	0.867608543	0.030947786

Lower			
Cultivar	Treatment	Mean Openboll Ratio	SE Open boll Ratio
D2020B3XF	No Spray	0.906009892	0.061317772
	Spray	0.987012987	0.008512486
DP2317B3TXF	No Spray	0.883608984	0.067762451
	Spray	0.930552781	0.044561794
DP2131B3TXF	No Spray	0.93986143	0.035533922
	Spray	0.95470573	0.024907966
24R6542B3TXF	No Spray	0.917663219	0.027653524
	Spray	0.875661356	0.038003704

Supplementary Table S6. Mean (± SE) of open boll ratio for each cotton cultivar under sprayed and no spray treatments during the growing season of 2025. Means were calculated across sampling dates during the squaring period.

Lower			
cultivar	Treatment	Mean Openboll Ratio	SE Open boll Ratio
24R6522B3TXF	No spray	0.774862548	0.029722423
	Spray	0.780942833	0.035991871
DP2020B3XF	No spray	0.80496633	0.05030185
	Spray	0.953846154	0.046153846
DP2131B3TXF	No spray	0.878323558	0.059286035
	Spray	0.862087363	0.065682144
DP2239B3XF	No spray	0.819640575	0.096920592
	Spray	0.811686062	0.046613312

Middle			
cultivar	Treatment	Mean Openboll Ratio	SE Open boll Ratio
24R6522B3TXF	No spray	0.718184651	0.067866923
	Spray	0.769137358	0.057405568
DP2020B3XF	No spray	0.801568104	0.050316983
	Spray	0.876516121	0.041717039
DP2131B3TXF	No spray	0.857041204	0.039456139
	Spray	0.822695917	0.064981786
DP2239B3XF	No spray	0.669272864	0.051685689
	Spray	0.741285831	0.060115433

Top			
cultivar	Treatment	Mean Openboll Ratio	SE Open boll Ratio
24R6522B3TXF	No spray	0.227775364	0.078714175
	Spray	0.271298793	0.09234426
DP2020B3XF	No spray	0.302562974	0.039714306
	Spray	0.432047197	0.085486784
DP2131B3TXF	No spray	0.396112336	0.068903908
	Spray	0.340752719	0.070407734
DP2239B3XF	No spray	0.142159105	0.040273803
	Spray	0.378655123	0.093987886

Supplementary Table S7. Mean (± SE) seed cotton weight from hand harvesting for each cotton cultivar under spray and no spray treatments during the growing season of 2024. Means were calculated across sampling dates during the squaring period.

Top			
Cultivar	Treatment	Mean Seed Cotton	SE Seed Cotton
D2020B3XF	No Spray	268.8697917	51.02230633
	Spray	268.8697917	40.52241649
DP2317B3TXF	No Spray	254.71875	31.00337893
	Spray	353.7760417	51.02230633
DP2131B3TXF	No Spray	311.3229167	60.70120622
	Spray	509.4375	79.04341708
24R6542B3TXF	No Spray	240.5677083	34.08022132
	Spray	382.078125	87.00301635

Middle			
Cultivar	Treatment	Mean Seed Cotton	SE Seed Cotton
D2020B3XF	No Spray	820.7604167	35.79961834
	Spray	1018.875	49.02064629
DP2317B3TXF	No Spray	778.3072917	55.53269754
	Spray	948.1197917	116.8639596
DP2131B3TXF	No Spray	891.515625	84.19572496
	Spray	976.421875	71.87837707
24R6542B3TXF	No Spray	735.8541667	132.7485377
	Spray	863.2135417	144.4515709

Lower			
Cultivar	Treatment	Mean Seed Cotton	SE Seed Cotton
D2020B3XF	No Spray	877.3645833	56.60416667
	Spray	919.8177083	110.5231686
DP2317B3TXF	No Spray	849.0625	72.70936858
	Spray	820.7604167	52.18646311
DP2131B3TXF	No Spray	849.0625	72.70936858
	Spray	764.15625	49.02064629
24R6542B3TXF	No Spray	834.9114583	51.02230633
	Spray	806.609375	29.00100545

Supplementary Table S8. Mean (± SE) seed cotton weight from hand harvesting for each cotton cultivar under spray and no spray treatments during the 2025 growing season. Means were calculated across sampling dates during the squaring period.

Top			
Cultivar	Treatment	Mean Seed Cotton	SE Seed Cotton
D2020B3XF	No Spray	866.04375	137.9563272
	Spray	1018.875	93.0101368
DP2239B3XF	No Spray	781.1375	90.65506753
	Spray	933.96875	89.05042627
DP2131B3TXF	No Spray	815.1	33.9625
	Spray	713.2125	20.79769885
24R6542B3TXF	No Spray	662.26875	77.81786352
	Spray	764.15625	93.0101368

Middle			
Cultivar	Treatment	Mean Seed Cotton	SE Seed Cotton
D2020B3XF	No Spray	1307.55625	83.19079539
	Spray	1460.3875	41.5953977
DP2239B3XF	No Spray	1375.48125	82.31966358
	Spray	1239.63125	99.01685186
DP2131B3TXF	No Spray	1375.48125	94.54759859
	Spray	1188.6875	46.5050684
24R6542B3TXF	No Spray	1273.59375	84.90625
	Spray	1341.51875	140.5448517

Lower			
Cultivar	Treatment	Mean Seed Cotton	SE Seed Cotton
D2020B3XF	No Spray	866.04375	67.925
	Spray	645.2875	78.73881704
DP2239B3XF	No Spray	747.175	82.31966358
	Spray	679.25	53.69942752
DP2131B3TXF	No Spray	849.0625	80.54914128
	Spray	730.19375	57.58622051
24R6542B3TXF	No Spray	866.04375	49.50842593
	Spray	1052.8375	102.5926122

Supplementary Table S9. Mean (± SE) seed cotton weight from machine harvesting for each cotton cultivar under spray and no spray treatments during the 2024 and 2025 growing season. Means were calculated across sampling dates during the squaring period.

Machine Harvesting 2024			
Cultivar	Treatment	Mean Seed Cotton	SE Seed Cotton
D2020B3XF	No Spray	2160.392361	84.48591945
	Spray	2415.111111	72.58685902
DP2317B3TXF	No Spray	1801.899306	136.6469343
	Spray	2198.128472	121.7684139
DP2131B3TXF	No Spray	1886.805556	134.4804032
	Spray	2377.375	65.36086173
24R6542B3TXF	No Spray	2000.013889	128.801258
	Spray	1820.767361	203.2589692

Machine Harvesting 2025			
Cultivar	Treatment	Mean Seed Cotton	SE Seed Cotton
D2020B3XF	No Spray	3676.721033	241.9207402
	Spray	4009.753634	309.8776361
DP2239B3XF	No Spray	3387.887203	187.8755339
	Spray	3544.124719	133.0655979
DP2131B3TXF	No Spray	4012.837269	224.5826095
	Spray	3569.821679	328.1758345
24R6542B3TXF	No Spray	4038.534229	284.8558722
	Spray	3596.546518	247.8306265
